# The lateral femoral notch sign and coronal lateral collateral ligament sign in magnetic resonance imaging failed to predict dynamic anterior tibial laxity

**DOI:** 10.1186/s12891-022-05368-9

**Published:** 2022-04-29

**Authors:** Tzu-Ching Huang, Zhao-Wei Liu, Chih-Kai Hong, Chi-Hsiu Wang, Kai-Lan Hsu, Fa-Chuan Kuan, Wei-Ren Su

**Affiliations:** 1 Department of Orthopaedic Surgery, National Cheng Kung University Hospital, College of Medicine, National Cheng Kung University, No.138, Sheng-Li Road, Tainan, Taiwan 70428; 2 Physical Therapy Center, National Cheng Kung University Hospital, College of Medicine, National Cheng Kung University, Tainan, Taiwan; 3 Skeleton Materials and Bio-compatibility Core Lab, Research Center of Clinical Medicine, National Cheng Kung University Hospital, College of Medicine, National Cheng Kung University, Tainan, Taiwan; 4 Department of Nursing, National Cheng Kung University Hospital, College of Medicine, National Cheng Kung University, Tainan, Taiwan; 5 Department of Biomedical Engineering, National Cheng Kung University, Tainan, Taiwan; 6 Musculoskeletal Research Center, Innovation Headquarter, National Cheng Kung University, Tainan, Taiwan

**Keywords:** GNRB, Notch sign, Coronal LCL sign, Dynamic anterior tibial laxity

## Abstract

**Purpose:**

To investigate the relationship between the lateral femoral notch sign as well as the coronal lateral collateral ligament (LCL) sign and anterior tibial translation using the GNRB arthrometer in patients with anterior cruciate ligament (ACL) injuries.

**Methods:**

Forty-six patients with ACL injuries were retrospectively included from May 2020 to February 2022; four patients were excluded due to incomplete data. Magnetic resonance imaging (MRI) were reviewed for the lateral femoral notch sign and the coronal LCL sign. The GNRB arthrometer was used to evaluate the dynamic anterior tibial translation of the knee, and the side-to-side differences (SSDs) in tibial translation between the injured knee and healthy knee were calculated at different force levels. Two types of slopes for displacement-force curves were acquired.

**Results:**

Six patients (14.3%) had the positive lateral femoral notch sign (notch depth > 2.0 mm), and 14 patients (33.3%) had the positive coronal LCL sign. The SSD of the anterior tibial translations under different loads as well as the slopes of displacement-force curves were the same in the positive and negative notch sign groups (*p* all > 0.05) and between the positive and negative coronal LCL sign groups (*p* all > 0.05). Meanwhile, the measured notch depth and notch length were also not significantly correlated with the anterior tibial translation SSD in the GNRB.

**Conclusion:**

The presence of the lateral femoral notch sign and the coronal LCL sign did not indicate greater dynamic tibial laxity as measured using the GNRB.

## Introduction

The occurrence of anterior cruciate ligament (ACL) injuries is high in certain sports, such as soccer, handball, and basketball [1]. Although some signs in conventional radiographs are helpful, magnetic resonance image (MRI) remains the best imaging modality for diagnosing an ACL tear [2]. In addition to changes in the integrity of the ACL itself, some additional findings in MRIs are not uncommon in patients with ACL tears, such as the lateral femoral notch sign [3], patellar and tibial edema [4], bone contusions on the femoral condyles and tibial plateau [5], and the coronal LCL sign [6].

The lateral femoral notch sign, defined as an impaction > 2 mm of the lateral femoral condyle, following an ACL injury is of special interest [2, 3]. Recently, it was found that the lateral femoral notch sign is correlated with concomitant posterior root tears of the lateral meniscus in patients with ACL tears [3]. However, little is known about whether the lateral femoral notch sign is a clinical indicator of greater dynamic anterior tibial laxity.

On the other hand, the coronal lateral collateral ligament (LCL) sign has been proposed to be correlated with an ACL-deficient knee with good interobserver and intraobserver reliabilities [6]. In studies conducted by Mitchell et al., the coronal LCL sign was suggested to be a marker for static laxity of the knee since greater anterior translation and internal rotation of the tibia were found [6, 7]. Although greater tibial anterior translation has been found with the presence of the coronal LCL sign, little is known about the correlation between the coronal LCL sign and dynamic anterior tibial laxity.

The purpose of the present study was to investigate the relationship between the lateral femoral notch sign as well as the coronal LCL sign and anterior tibial translation using the GNRB arthrometer in patients with ACL injuries. We hypothesized that the lateral femoral notch sign and the coronal LCL sign would reflect greater anterior tibial laxity.

## Materials and methods

### Patients

This study was a retrospective review performed in a tertiary medical center. After approval from the Institutional Review Board (IRB) at the National Cheng Kung University Hospital (A-ER-110-437), a retrospective review was performed on a cohort of all patients diagnosed with ACL tears at the authors’ institution between May 2020 and February 2022. Patients who had completed both an MRI and a GNRB arthrometer examination (Genourob, Laval, France) were included. Both exams were performed within 6 months after the injury. Patients with low resolution MRI, incomplete data from the chart review, previous ipsilateral knee ligament injuries or surgeries, and previous contralateral knee injury or surgeries were excluded. Relevant patient characteristics, including age, gender, and laterality, were collected and analyzed retrospectively.

### Image analysis

The lateral femoral notch sign and the coronal LCL sign were evaluated from the patients’ MRI. The evaluations of these signs were performed separately by two operators (T-C H and C-K H) twice with a two-week interval between the evaluations. The intra- and inter-observer reliability were assessed by calculating the intra-class correlation coefficients (ICCs). ICC values less than 0.5, between 0.5 and 0.75, between 0.75 and 0.9, and greater than 0.90 are indicative of poor, moderate, good, and excellent reliability, respectively [8]. Imaging measurements were performed via a picture archiving and communication system (INFINITT PACS, INFINITT Healthcare Co. Ltd., South Korea).

**Fig. 1:**
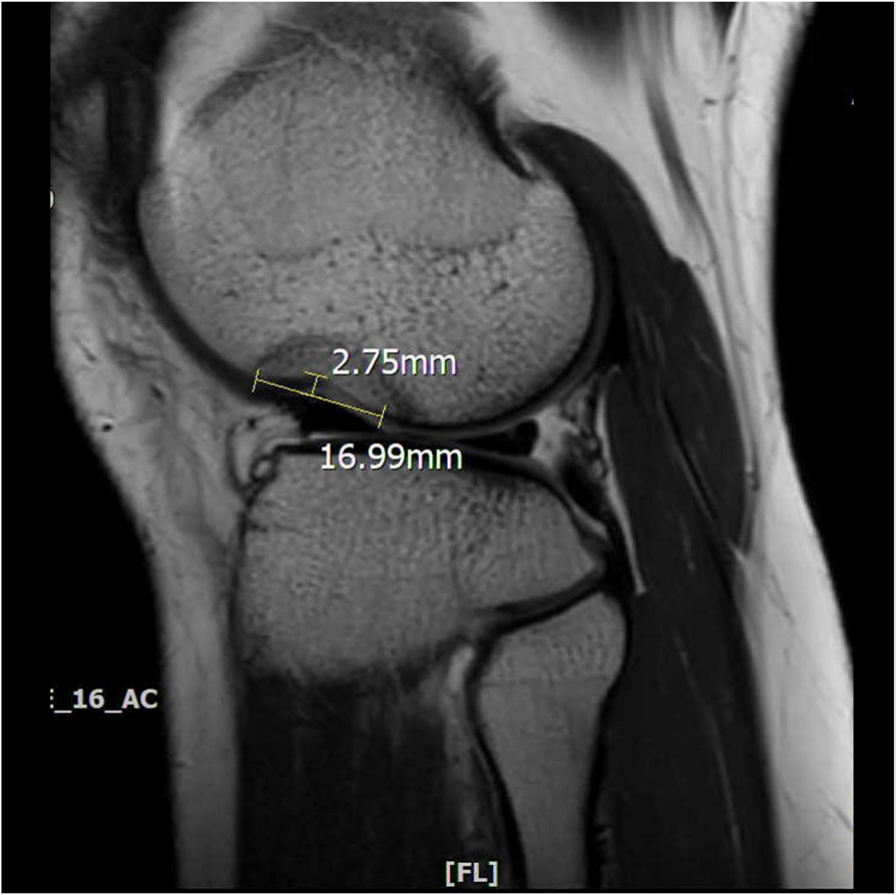
Measurement of the lateral femoral notch sign. The notch sign was measured on a sagittal T1-weighted magnetic resonance imaging based on the tangent method following a previous study [3]

**Fig. 2:**
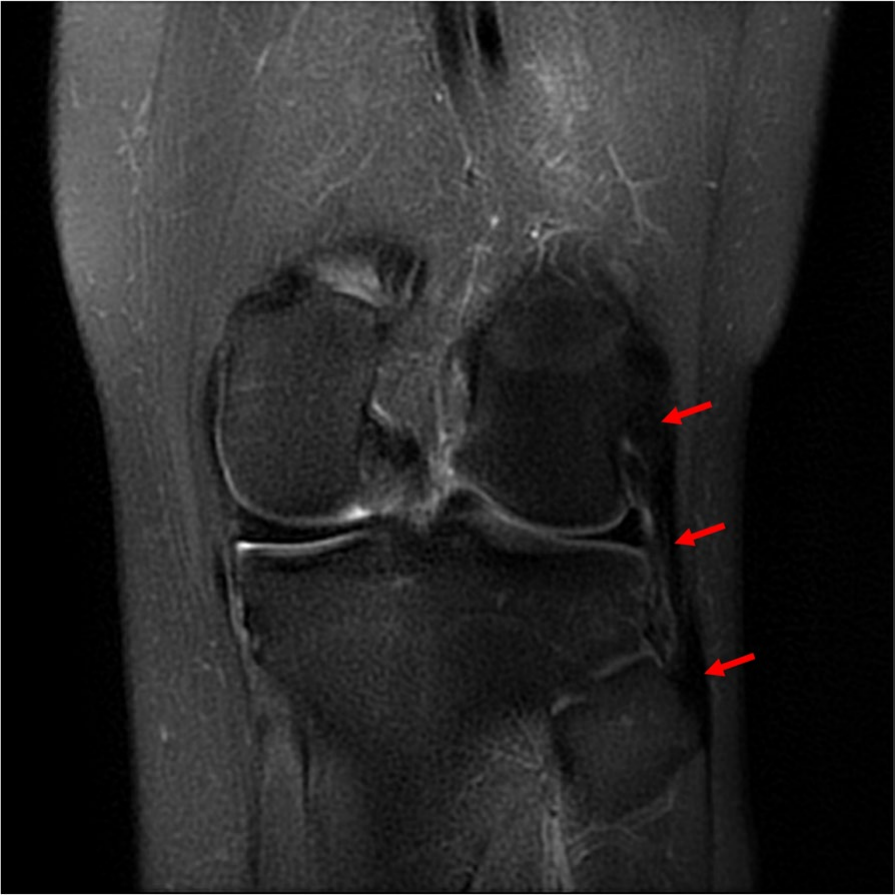
Positive coronal LCL sign (red arrows) in magnetic resonance imaging

The lateral femoral notch sign was identified on a T1-weighted sagittal MRI. The notch depth was measured as the distance between the deepest portion of the sulcus and the tangent line connecting the anterior and posterior edge of the lateral femoral notch [3] (Fig. 1). Based on previous studies [2, 3, 9, 10], a notch sign was considered to be positive if the notch depth was greater than 2.0 mm. A positive notch sign was further categorized into grade 1 (2.0 mm to 3.9 mm) and grade 2 (≥4.0 mm) [2]. We also measured the length of the notch sign, defined as the length of the tangent line between the anterior and posterior of the lateral femoral notch. The coronal T2 image or proton density image slices were reviewed on the MRI for the coronal LCL sign. The coronal LCL sign was considered positive when the LCL could be observed entirely from the femoral origin to the fibular insertion in a single coronal slice on the MRI image (Fig. 2) [6, 7].

### Knee anterior tibial translation measurement

**Fig. 3:**
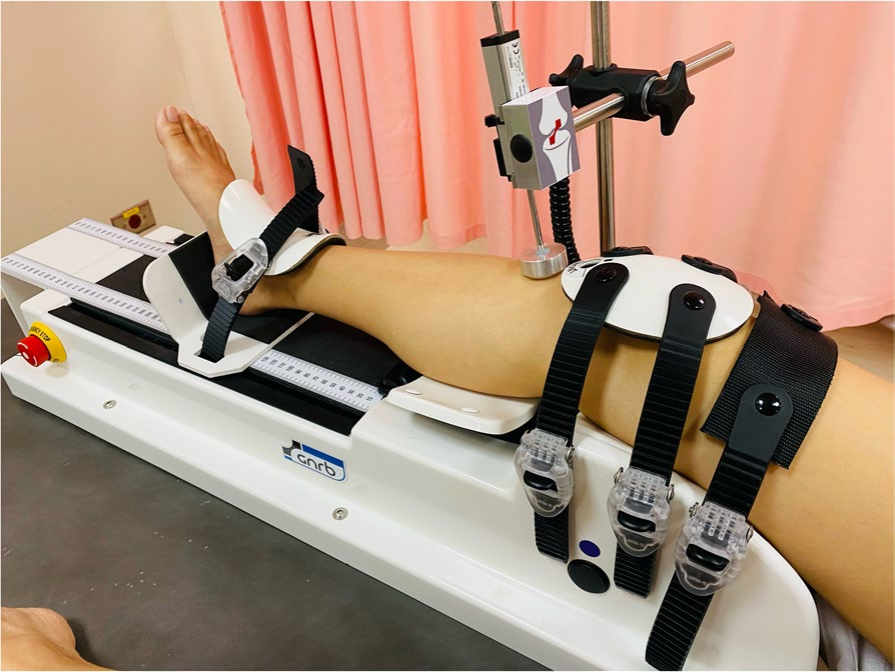
The setup of the GBRB arthrometer system

The GNRB arthrometer (displacement in millimeters, with a 0.1 mm accuracy) was used to evaluate knee laxity by measuring the anterior tibial translation (Fig. 3). In accordance with the recommendations of the manufacturer of the GNRB (Genourob, Laval, France), the knee was placed in 20° flexion at a 0° rotation in a molded support in order to reproduce the typical Lachman test position [11, 12]. The data were automatically collected in the computer. The examinations were performed for both knees, and the side-to-side difference (SSD) between the injured knee and the healthy knee was calculated at each force level (89, 134, 150, and 200 N) for each subject with a 0.1 mm accuracy. For each applied force, the anterior tibial translation was measured simultaneously using the navigation system (3 measurements per knee). The displacement-force curve was created, and the slope of the curve was calculated. Slope 1 (S1) was defined as the slope of the curve ranging between 0 and 100 N, whereas slope 2 (S2) defined as the slope of the curve ranging between 100 N and the maximum force. The SSDs in S1 and S2 between the injured knee and healthy knee were calculated for the analysis.

### Statistical analysis

All statistical analyses were conducted using SPSS Statistics version 20 software (IBM SPSS Inc., Chicago, IL, USA). Descriptive statistics, including means and standard deviations were obtained. A chi-square test was used for the purpose of comparing the between-group categorical data. The Mann-Whitney U test was conducted to compare the between-group parameters. The Spearman correlation was used to evaluate the relationship between nonparametric variables. Statistical significance was set as *p* ≤ 0.05. Post-hoc power analyses were performed with G*Power Version 3.1.3 (Heinrich Heine-University of Dusseldorf, Dusseldorf, Germany) to calculate the achieved power. An alpha equal to 0.05 was given.

## Results

### Demographic data

Forty-six patients were initially included in the study. Four patients were excluded due to incomplete data, and 42 patients were finally enrolled for the analysis. The basic characteristics were summarized in (Table 1). Six patients (14.3%) had the positive lateral femoral notch sign, the average notch depth and notch width of which were 2.30 ± 0.2 mm and 14.7 ± 2.9 mm, respectively. All these positive notch signs were graded as grade 1 notch signs. Meanwhile, 14 patients (33.3%) in the study had positive coronal LCL signs. The mean age and gender distribution were the same for the aforementioned subgroups (Table 1). The interobserver repeatability and intraobserver repeatability of notch width, notch depth and LCL signs were summarized in Table 2.

**Table 1:** Basic characteristics of the different notch sign groups and the coronal LCL sign groups

	Age (year, mean ± SD)	Gender
All patients	26.7 ± 8.5	28 male, 14 female
Notch sign (+) (*n* = 6)	24.2 ± 6.4	4 male, 2 female
Notch sign (−) (*n* = 36)	27.1 ± 8.8	24 male, 12 female
*p* value	0.516	0.689
LCL sign (+) (*n* = 14)	26.1 ± 8.1	9 male, 5 female
LCL sign (−) (*n* = 28)	27.0 ± 8.8	19 male, 9 female
*p* value	0.830	0.541

**Table 2:** The interobserver repeatability and intraobserver repeatability of notch width, notch depth and the coronal LCL sign

	Intraobserver repeatability of researcher 1	Intraobserver repeatability of researcher 2	Interobserver repeatability
Notch depth	0.95	0.96	0.95
Notch width	0.82	0.83	0.81
LCL sign	0.89	0.93	0.86

### Dynamic anterior tibial translation

The anterior tibial translation under different loads and slopes of the displacement-force curve from the GNRB for the different notch sign groups and the coronal LCL sign groups are summarized in Table 3. The SSDs of an anterior tibial translation under 134 N was the same for the positive notch sign group (3.8 ± 1.4 mm) and the negative notch group (4.0 ± 1.8 mm) (*p* = 0.875). Meanwhile, the SSD for S2 from the GNRB displacement-force curve was the same for the positive notch sign group (12.5 ± 8.9 mm/N) and the negative notch group (9.3 ± 6.8 mm/N) (*p* = 0.449). On the other hand, the SSDs in the anterior tibial translation under 134 N were the same for the positive coronal LCL sign group (3.9 ± 1.9 mm) and the negative coronal LCL sign group (4.0 ± 1.7 mm) (*p* = 0.722). The SSD of S2 from the GNRB displacement-force curve was the same for the positive coronal LCL sign group (10.7 ± 6.7 mm/N) and the negative coronal LCL sign group (9.2 ± 7.3 mm/N) (*p* = 0.348). The calculated effect sizes for these data ranged from 0.61 to 0.74. With a given α equal to 0.05, the post hoc achieved powers ranged from 48 to 61%.

**Table 3:** Mean anterior tibial translation and slope of displacement-force curve for the different notch sign groups and the coronal LCL sign groups

	Anterior tibial translation SSD (mm, mean ± SD)			Slope of displacement-force curve SSD (mm/N, mean ± SD)	
	134 N	150 N	200 N	S1	S2
Notch sign (+)	3.8 ± 1.4	4.0 ± 1.5	4.6 ± 1.5	30.1 ± 18	12.5 ± 8.9
Notch sign (−)	4.0 ± 1.8	4.1 ± 1.9	4.4 ± 2.1	40.5 ± 25	9.3 ± 6.8
*p* value	0.875	0.999	0.875	0.388	0.449
LCL sign (+)	3.9 ± 1.9	4.0 ± 2.0	4.3 ± 2.1	38.6 ± 27	10.7 ± 6.7
LCL sign (−)	4.0 ± 1.7	4.1 ± 1.8	4.5 ± 2.0	39.4 ± 23	9.2 ± 7.3
*p* value	0.722	0.742	0.722	0.823	0.348

*SSD* side-to-side difference

There were no significant correlations between the anterior tibial translation SSD under different loads in the GNRB and the measured notch depth as well as notch width. Meanwhile, the notch depth and notch width were also not found to be correlated with the SSDs of S1 and S2 from the GNRB displacement-force curve. The detailed data are summarized in Table 4.

**Table 4:** The Spearman correlation between the parameters measured from the GNRB and the measured notch depth and notch width

	Anterior tibial translation SSD			SSD of S1	SSD of S2
	134 N	150 N	200 N		
Notch Depth	0.018	0.035	0.080	−0.159	0.174
*P* value	0.909	0.826	0.614	0.316	0.270
Notch Width	−0.016	0.002	0.035	−0.171	0.248
*P* value	0.920	0.988	0.826	0.279	0.114

*SSD* side-to-side difference

## Discussion

The major finding of the current study was that there were no significant differences in parameters measured from the GNRB between the positive and negative notch sign groups or between the positive and negative coronal LCL sign groups. The lateral femoral notch sign, which is associated with concomitant posterior root tears in patients with ACL injuries [3], potentially leads to greater knee instability [13, 14]. Meanwhile, the greater static tibial anterior translation has been found in patients with ACL injuries with the positive coronal LCL sign [6, 7]. The present study further investigated whether the presence of the lateral femoral notch sign and the coronal LCL sign lead to greater dynamic anterior tibial laxity.

No correlation between the coronal LCL sign and the dynamic tibial anterior translation was found in the present study. The coronal LCL sign is a new concept. Previous studies have indicated that patients with the positive coronal LCL sign have greater tibia anterior translation in the resting position [6, 7]. Since the findings from the images represent static laxity of the knee, the present study was aimed toward further evaluation of the correlation between the coronal LCL sign and anterior tibial laxity using the GNRB arthrometer. Different from the hypothesis, the results suggested that the coronal LCL sign had limited predictive in terms of dynamic anterior tibial laxity.

The lateral femoral notch sign in MRI is not uncommon, and its clinical relevance is still under investigation. A recent study in patients with ACL tears reported that identifying the lateral femoral notch sign in an MRI may be predictive of concomitant posterior root tears of the lateral meniscus [3], which has been reported to be related to greater anterior tibia translation during the pivot-shift test [13, 14] and a trend toward greater tibial translation during the Lachman test [14]. The current study provided a direct evaluation of the correlation between the lateral femoral notch sign and anterior tibial laxity using the GNRB arthrometer. Different from the hypothesis made in the study, the results suggested that the use the lateral femoral notch sign alone without identifying other structural injuries failed to predict dynamic anterior laxity of knee. Although the lateral femoral notch sign was not related to dynamic anterior knee laxity, it was reported to be correlated with increased rotatory laxity after ACL injury [15]. Therefore, identifying the lateral femoral notch sign remains clinically valuable.

Although the data from different studies could not be compared directly, the data acquired in the present study were generally consistent with those in the previous studies on this topic. The prevalence of the notch sign in our study group was 14.3% (6/42), which was compatible with that found in the previous studies, ranging from 3.2 to 52% [2, 15–19]. The interobserver and intraobserver reliability values were also similar, where in previous studies, they were 0.91 and 0.90 to 0.94, respectively [15, 16], and in the present study, they were 0.86 and 0.89 /0.93, respectively.

There were several limitations in the study. First, this is a retrospective study which made it difficult to obtain data. However, we managed to obtain all the data and basic characteristics for the study. Selection bias is also common drawback of a retrospective study. Secondly, our sample sizes and the achieved powers in the analyses were relatively small. As the present study acted like a pilot study, future studies on the related topics with larger sample sizes or collaboration studies from different departments will be warranted in order to provide sufficient statistical power [20]. Third, the quality of the MRI could not be fully controlled since the study was a retrospective design. The majority of MRIs were acquired in a consistent position, the supine position with the knee at 10° to 15° of flexion and a neutral rotation, even if positioning inconsistencies possibly existed due to < 10% of images being obtained at an outside facility. Fourth, the presence of concomitant soft tissue injuries was not evaluated in the present study. Instead, the relationship between the included signs and dynamic anterior tibial laxity were directly evaluated.

## Conclusion

The presence of the lateral femoral notch sign and the coronal LCL sign did not indicate greater dynamic tibial laxity as measured using the GNRB.

## Data Availability

The datasets used and/or analysed during the current study are available from the corresponding author on reasonable request.

## References

[1] Mouton C, Gokeler A, Urhausen A, Nuhrenborger C, Seil R. High incidence of anterior cruciate ligament injuries within the first 2 months of the season in amateur team ball sports. Sports Health. 2022;14(2):183–7. 10.1177/19417381211014140 PMC8883411 34039120

[2] Herbst E, Hoser C, Tecklenburg K, Filipovic M, Dallapozza C, Herbort M (2015). The lateral femoral notch sign following ACL injury: frequency, morphology and relation to meniscal injury and sports activity. Knee Surg Sports Traumatol Arthrosc.

[3] Berthold DP, Muench LN, Herbst E, Mayr F, Chadayammuri V, Imhoff AB (2021). High prevalence of a deep lateral femoral notch sign in patients with anterior cruciate ligament (ACL) and concomitant posterior root tears of the lateral meniscus. Knee Surg Sports Traumatol Arthrosc.

[4] Wissman RD, England E, Mehta K, Nepute J, Von Fischer N, Apgar J (2014). Patellotibial contusions in anterior cruciate ligament tears. Skelet Radiol.

[5] Yoon KH, Yoo JH, Kim KI (2011). Bone contusion and associated meniscal and medial collateral ligament injury in patients with anterior cruciate ligament rupture. J Bone Joint Surg Am.

[6] Mitchell BC, Siow MY, Bastrom T, Bomar JD, Pennock AT, Parvaresh K (2021). Coronal lateral collateral ligament sign: a novel magnetic resonance imaging sign for identifying anterior cruciate ligament-deficient knees in adolescents and summarizing the extent of anterior Tibial translation and Femorotibial internal rotation. Am J Sports Med.

[7] Mitchell BC, Siow MY, Bastrom T, Bomar JD, Pennock AT, Parvaresh K (2021). Predictive value of the magnetic resonance imaging-based coronal lateral collateral ligament sign on adolescent anterior cruciate ligament reconstruction graft failure. Am J Sports Med.

[8] Koo TK, Li MY (2016). A guideline of selecting and reporting Intraclass correlation coefficients for reliability research. J Chiropr Med.

[9] Hoffelner T, Pichler I, Moroder P, Osti M, Hudelmaier M, Wirth W (2015). Segmentation of the lateral femoral notch sign with MRI using a new measurement technique. BMC Musculoskelet Disord.

[10] Kanakamedala AC, Burnham JM, Pfeiffer TR, Herbst E, Kowalczuk M, Popchak A (2018). Lateral femoral notch depth is not associated with increased rotatory instability in ACL-injured knees: a quantitative pivot shift analysis. Knee Surg Sports Traumatol Arthrosc.

[11] Pouderoux T, Muller B, Robert H (2020). Joint laxity and graft compliance increase during the first year following ACL reconstruction with short hamstring tendon grafts. Knee Surg Sports Traumatol Arthrosc.

[12] Saravia A, Cabrera S, Molina CR, Pacheco L, Munoz G (2020). Validity of the Genourob arthrometer in the evaluation of total thickness tears of anterior cruciate ligament. J Orthop.

[13] Frank JM, Moatshe G, Brady AW, Dornan GJ, Coggins A, Muckenhirn KJ (2017). Lateral meniscus posterior root and Meniscofemoral ligaments as stabilizing structures in the ACL-deficient knee: a biomechanical study. Orthop J Sports Med.

[14] Shybut TB, Vega CE, Haddad J, Alexander JW, Gold JE, Noble PC (2015). Effect of lateral meniscal root tear on the stability of the anterior cruciate ligament-deficient knee. Am J Sports Med.

[15] Lucidi GA, Grassi A, Di Paolo S, Agostinone P, Neri MP, Macchiarola L (2021). The lateral femoral notch sign is correlated with increased rotatory laxity after anterior cruciate ligament injury: pivot shift quantification with a surgical navigation system. Am J Sports Med.

[16] Dimitriou D, Reimond M, Foesel A, Baumgaertner B, Zou D, Tsai TY (2021). The deep lateral femoral notch sign: a reliable diagnostic tool in identifying a concomitant anterior cruciate and anterolateral ligament injury. Knee Surg Sports Traumatol Arthrosc.

[17] Fayad LM, Parellada JA, Parker L, Schweitzer ME (2003). MR imaging of anterior cruciate ligament tears: is there a gender gap?. Skelet Radiol.

[18] Garth WP, Greco J, House MA (2000). The lateral notch sign associated with acute anterior cruciate ligament disruption. Am J Sports Med.

[19] Yu JS, Bosch E, Pathria MN, McAndless M, Mishra D, Daniel D (1995). Deep lateral femoral sulcus: study of 124 patients with anterior cruciate ligament tear. Emerg Radiol.

[20] Mehler DMA, Edelsbrunner PA, Matić K (2019). Appreciating the significance of non-significant findings in psychology. J Eur Psychol Stud.

